# The effect of choline-stabilized orthosilicic acid in patients with peri-implantitis: an exploratory randomized, double-blind, placebo controlled study

**DOI:** 10.1186/s12903-021-01817-4

**Published:** 2021-09-29

**Authors:** Wim Teughels, Gizem Unal Celik, Mihai Tarce, Ine De Cock, Sara M. Persyn, Mehmet C. Haytac

**Affiliations:** 1 Section of Periodontology, Department of Oral Health Sciences, KU Leuven and Dentistry, University Hospitals, Leuven, Belgium; 2 Department of Periodontology, Faculty of Dentistry, Cukurova University, Adana, Turkey; 3 Research and Development, Bio Minerals NV, Zenderstraat 12, 9070 Destelbergen, Belgium

**Keywords:** Choline-stabilized orthosilicic acid, Peri-implantitis, Bone, Mucosal recession

## Abstract

**Background:**

Choline-stabilized orthosilicic acid (CS-OSA) was previously found to stimulate bone collagen formation in osteopenia and to improve biomarkers of cartilage degradation in knee osteoarthritis. The aim of the present study was to investigate the effect of oral administration of CS-OSA on clinical symptoms of peri-implantitis and the associated bone loss.

**Methods:**

Twenty-one patients with peri-implantitis were randomized in CS-OSA or placebo groups. After initial clinical and cone beam computed tomography (CBCT) measurements [probing pocket depth (PPD), bleeding on probing (BOP), mucosal recession (REC), distance from implant shoulder to alveolar crest (IS-AC) and distance from implant shoulder to first bone-to-implant contact (IS-BIC)], flap operations were performed at the peri-implantitis sites. All patients were instructed to use either placebo or CS-OSA capsules twice a day for 1 year. Measurements were repeated 6 and 12 months after randomization.

**Results:**

The data of 18 patients (36 implants) were used in the per protocol analysis. PPD and BOP improved significantly (*p* < 0.05) compared to baseline for both groups after 6 and 12 months. However, REC significantly increased in the placebo group but not in the CS-OSA group. The change in REC over 6 and 12 months was significantly different between groups (*p* < 0.01). IS-BIC and IS-AC measurements remained stable in the CS-OSA group whereas in the placebo group, both parameters increased significantly after 6 and 12 months. The change in IS-BIC over 12 months was significantly different between groups (p < 0.05).

**Conclusion:**

The results of this preliminary study suggest that CS-OSA may stabilize and even prevent further bone loss after surgical peri-implantitis treatment and support mucosal tissue healing.

*Trial registration*

The trial was retrospectively registered at ISRCTN registry, registration number: ISRCTN14348802, registration date: 24/06/2020.

**Supplementary Information:**

The online version contains supplementary material available at 10.1186/s12903-021-01817-4.

## Background

Dental implants have been widely used for the replacement of missing teeth since the 1980’s [1]. High long-term survival rates of over 95% have been reported [2]. However, the prevalence of peri-implant diseases affecting both soft and hard tissues that may eventually lead to implant failure (loss) has substantially increased to up to more than 20% in the past decade [1–4]. Appropriate treatment of implants affected by peri-implant diseases is becoming increasingly important as the number of implants placed per year continues to increase [5].

Peri-implantitis is a plaque-associated pathological condition occurring in tissues around dental implants, characterized by inflammation in the peri-implant mucosa and subsequent progressive loss of supporting bone [6]. The diagnosis and follow-up of peri-implantitis are made by detecting radiographic bone loss, clinical signs of inflammation, bleeding on probing (BOP) with or without suppuration, increased probing pocket depth (PPD) and recession of the mucosal margin (REC) [6, 7]. Based on the consensus report of Workgroup 4 of the 2017 World Workshop on the Classification of Periodontal and Peri-Implant Diseases and Conditions, bone levels of ≥ 3 mm apical of the most coronal portion of the intra-osseous part of the implant together with bleeding on probing are consistent with the diagnosis of peri-implantitis in studies [6]. Intraoral radiography (IR) is the most commonly used technique for the measurement of peri-implant bone loss [8]. However, IR is a two-dimensional (2D) imaging technique offering merely mesiodistal and vertical detection of bone defects [9]. Therefore cone beam computed tomography (CBCT) has been widely used for three-dimensional (3D) assessment offering additional spatial information including buccolingual visualization of the peri-implant bone [10].

The main goals of peri-implantitis treatment are resolving inflammation and preventing further bone loss by decontaminating the implant surface. Non-surgical treatment by mechanical debridement with or without antiseptic and/or antibiotic therapy has shown limited success [11, 12]. The surgical treatment options including flap surgery with or without osseous resection, regeneration with bone grafts and guided bone regeneration are proven to be more effective [5, 12]. However until now, a gold standard treatment for peri-implantitis is lacking [12].

Choline-stabilized orthosilicic acid (CS-OSA), is a concentrated and stable complex of choline and orthosilicic acid [13]. Physiological concentrations of orthosilicic acid were found to stimulate the synthesis of collagen type I in human osteoblast-like cells and skin fibroblasts [14]. CS-OSA supplementation of animals resulted in an increased femoral bone density [15, 16]. This effect was confirmed in humans demonstrating a beneficial effect on bone turnover, especially on bone collagen formation and femoral bone mineral density, when treating osteopenic women with CS-OSA and Ca/Vit D3 for 12 months compared with Ca/Vit D3 alone [17]. A possible effect of CS-OSA on collagen metabolism was also suggested when photoaged women were found to have improved surface and mechanical properties of the skin when taking CS-OSA compared to women who took a placebo [18]. Recently, 12-week CS-OSA supplementation of men with knee osteoarthritis resulted in symptomatic improvements associated with a significant reduction of cartilage degradation biomarkers, suggesting a possible effect of CS-OSA on collagen metabolism in both cartilage and subchondral bone [19].

Based on these previous studies [13–19], one can hypothesize that CS-OSA may have a possible effect on bone loss in peri-implantitis. The aim of this explorative study was to evaluate the effect of the oral intake of CS-OSA over a 12-month period on clinical symptoms of peri-implantitis and the associated bone loss.

## Methods

### Patients

A 12-month, randomized, double-blind placebo-controlled parallel-group study was performed in patients with peri-implantitis. Ethical approval was obtained from the local Ethical Committee of the Cukurova University, Adana, Turkey (65/4, 04.05.2017). The study was conducted in accordance with the Helsinki Declaration as revised in 2013 and was retrospectively registered at ISRCTN registry with registration number: ISRCTN14348802 and registration date: 24/06/2020.

Patients consulting the Department of Periodontology of the dental school of the Cukurova University were screened for participating in the study between September 2017 and January 2018. The inclusion criteria defined eligible patients as men and women between 18 and 75 years of age with single or multiple osseointegrated implants suffering from peri-implantitis which was defined as bone loss of more than 3 mm measured on intra-oral radiographs and a PPD of more than 4 mm with BOP or suppuration on probing. All patients gave written informed consent.

The exclusion criteria were the following: pregnancy or breastfeeding, smoking or history of smoking (less than 6 months prior to the start of the study), recent or current alcohol and drug abuse, gingival index score > 2 [20], active, untreated periodontitis, mobile implants, poorly controlled diabetes (Glycated hemoglobin (HbA1c) level > 6%), osteonecrosis of the jaw and participation in another clinical trial. Furthermore, patients with renal failure, documented history of stroke, myocardial infarct or cancer and patients belonging to a high risk group for HIV were excluded. Additionally, specified concomitant and previous medications (i.e. dietary supplements containing a silicon source, systemic antibiotics, bisphosphonates, local antiseptics) that could interfere with the outcome of the study were prohibited during the trial. A wash-out period of 3 months and 6 months was required for the use of food supplements containing silicon sources and systemic antibiotics, respectively, prior to the baseline visit.

### Patient visits

During a first visit, the screening visit, patients were screened for the inclusion and exclusion criteria. If needed, a wash-out period was required. At the baseline visit (T0), baseline assessments were performed by clinical parameters evaluation, OHIP-14 questionnaire completion, radiographic analysis and biochemical analysis. Surgery was performed within a period of 10 days after the baseline visit. The sutures were removed 10 days after surgery. Assessments were repeated 6 and 12 months after the baseline visit (T6, T12). The visits at 2, 4, 8 and 10 months after baseline were performed to check for adverse events, oral hygiene condition and study medication compliance and to motivate the patient to continue with the study. All patients received proper oral hygiene instructions and were given the same tooth brush (Oral B Pro-flex Clinical Line), tooth paste (Oral B Ipana Pro-expert Clinical Line) and interdental brush (Oral B Interdental 228 mm) at the baseline visit. During each follow-up visit (2-monthly), the oral hygiene condition of each patient was evaluated, a professional prophylaxis was performed if needed, oral hygiene instructions were repeated and the tooth brush, tooth paste and interdental brush were replaced to standardize the maintenance procedures for each patient.

### Study medication and randomization

Twenty-one patients were randomly assigned to take a capsule of either the active treatment (520 mg beadlets containing 5 mg of silicon and 100 mg of choline in the form of CS-OSA (ch-OSA®, Bio Minerals NV, Belgium [18])) or placebo (520 mg microcrystalline cellulose beadlets; Pharmatrans Sanaq AG, Switzerland) twice daily, one in the morning and another in the evening with a glass of water or juice, for 12 months. The treatment allocation occurred sequentially in a 1:1 ratio using a randomization list, which was generated by an independent statistician in R (software version 3.3.3 for Windows; The R Foundation for Statistical Computing, Vienna, Austria). More specifically, block randomization was used in randomly selected block sizes of 2 or 4. The individual code was kept in a sealed envelope by the investigator, who was instructed not to open this except in case of medical emergency.

At baseline and after 2, 4, 6, 8 and 10 months the study medication was delivered in bottles labeled with the patient’s randomization number (according to the allocation sequence). Blinding among patients, investigators and monitors was maintained by providing identical packaging, appearance, taste and odor for CS-OSA and placebo capsules, respectively. Treatment compliance was verified at subsequent visits by counting the number of unused capsules.

### Assessments and outcome measures

During screening, clinical parameters including PPD, BOP (present = 1, absent = 0) and REC (relative to the implant abutment junction) were measured at six sites per implant with a calibrated probe (North Carolina periodontal probe, Hu-Friedy, Chicago, IL, USA). An IR (i.e. less than 3 months old) was used to determine the bone loss at the peri-implantitis sites. All initial and post-operative clinical measurements were carried out by a single examiner (M.C.H.) who was blinded to the study groups. A prior measurement of PPD in 20 implants with peri-implantitis (not included in the study) was repeated three times for intra-examiner calibration resulting in an intra‐agreement coefficient of κ = 0.86. Evaluation of clinical parameters was repeated at baseline and at 6 and 12 months after randomization by the same clinician that performed the measurements during screening. Additionally a Patient’s Oral Health related Quality of Life questionnaire (OHIP-14) was completed by each patient, blood samples were taken and a CBCT was performed at these visits.

#### OHIP-14 questionnaire

The OHIP-14 questionnaire (a Turkish translation of Başol et al. [21], was used in the present study), is a validated questionnaire containing 14 questions specifically designed for patients with dental problems [22]. The questionnaire was self-completed by the patients at baseline and at 6 and 12 months. The frequency of each symptom is scored on a 5-point scale, ranging from never (score 0), hardly ever (score 1), occasionally (score 2), fairly often (score 3) and very often (score 4). The scores are summed to yield a total OHIP score (range 0–56), with higher scores on the OHIP being indicative of more inconvenience in daily life.

#### Radiographic analysis

**Fig. 1:**
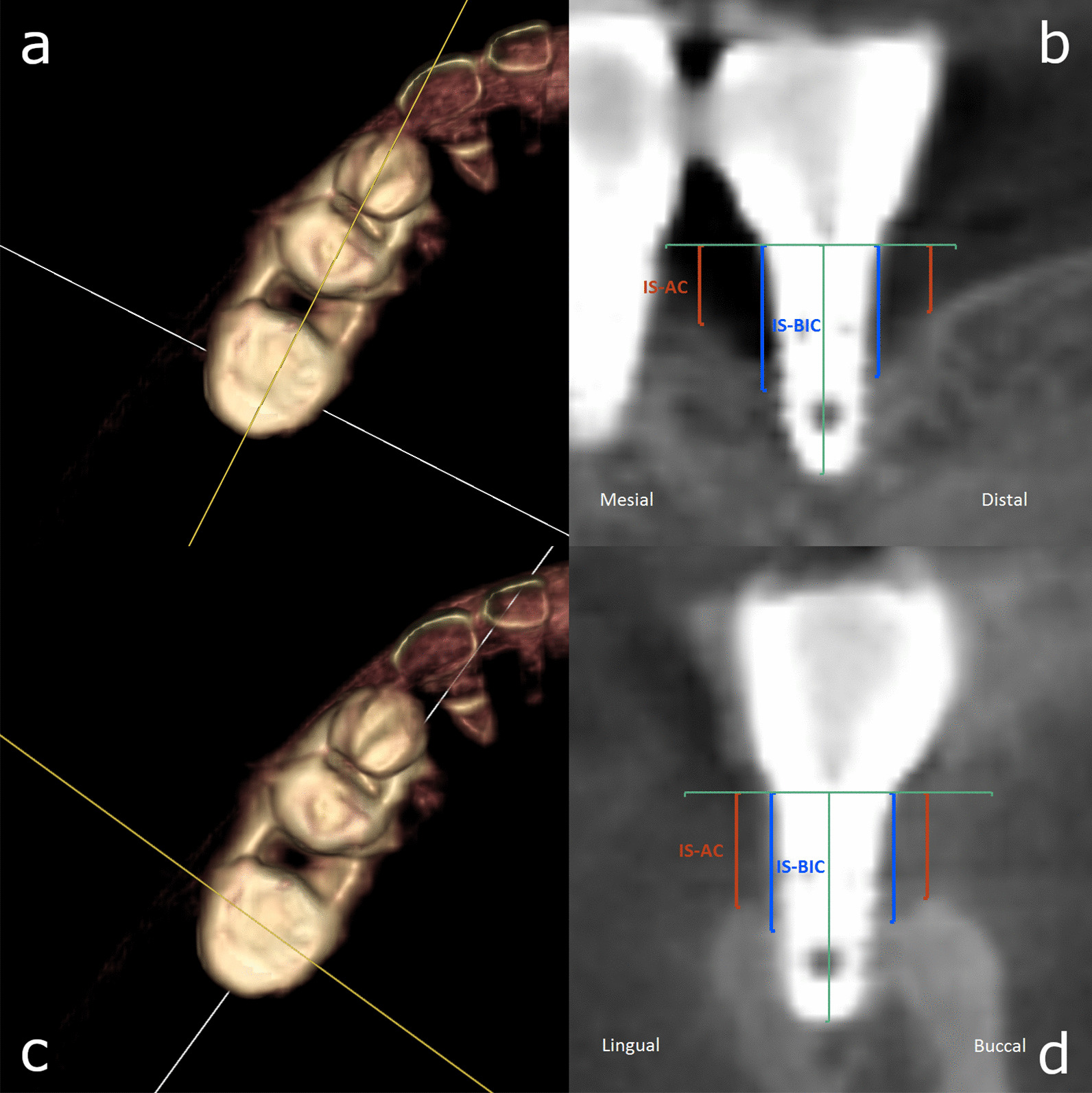
Right images (**b**, **d**): Cone beam computed tomography (CBCT) slices illustrating the measured parameters: distance from implant shoulder to first bone-to-implant contact (IS-BIC; blue) and distance from implant shoulder to alveolar crest (IS-AC; red) at the lingual, buccal, mesial and distal aspects of the implants. The level of the implant shoulder (IS) and the long axis of the implant are marked in green. Left images (**a**, **c**): 3D volume rendering of the jaw and the cross-sections (yellow plane) made to perform the measurements illustrated in the images on the right. The measurements were performed on two perpendicular planes passing through the long axis of each implant and oriented mesio-distally and bucco-orally, respectively

CBCT scans were taken with the Planmeca ProMax 3D Mid (Helsinki, Finland) at baseline and at 6 and 12 months. The scans were made with the following technical parameters: 90 kV, 10 mA, a field of view area of 45 × 45 × 43,6 mm, a voxel size of 75 µm and a scanning time of 27 s. The CBCT scans were registered using a voxel-based algorithm (Amira 2019.1, Thermofisher Scientific) as described previously [23]. Measurements were then performed using the open-source 3D Slicer 4.10 software program [24]. Cross-sectional slices were created through the long axis of each implant to measure the distance (mm) from the implant shoulder to the alveolar crest (IS-AC) and the distance (mm) from the implant shoulder to the first bone-to-implant contact (IS-BIC) at the buccal, lingual, mesial and distal aspects (four sites per implant) (Fig. 1) [25]. The investigator was blinded while evaluating these parameters.

#### Biochemical analysis

Finally, fasting serum was collected to assess biochemical markers of bone formation, bone resorption and inflammation at baseline, 6 months and 12 months. For the collection of serum, Vacuette® serum tubes (Greiner Bio-One) were used. Aliquots of the samples were stored at −20 °C until analysis. More specifically, C-terminal telopeptide of collagen type I (CTX-I), bone alkaline phosphatase (BAP), osteocalcin, N-terminal propeptide of type I procollagen (PINP) and high sensitive C-reactive protein (hsCRP) concentrations were determined using the serum Crosslaps ELISA from IDS (UK), the Ostase BAP from IDS (UK), the hOST-EASIA from Diasource (Belgium), the UniQ PINP RIA from Orion Diagnostica (Finland) and the Liquid Unassayed Multiqual from Bio-Rad Laboratories (USA), respectively.

#### Surgical procedure

All surgical procedures were performed by the same experienced periodontist (G.U.C.) who was blinded to the study groups. After local anesthesia, sulcular incisions were made and full-thickness mucoperiosteal flaps were raised to expose the implant surfaces. Inflammatory tissue was removed, and the implant surfaces were mechanically cleaned with titanium-coated curettes and chemically disinfected with EDTA gel. Then the soft tissue flaps were sutured with 5–0 sutures. Bone grafts, membranes or other biologics were placed in neither of the two treatment groups (placebo and CS-OSA).

#### Outcome measures

The primary outcome measure was the change in PPD at peri-implantitis sites from baseline to 12 months. Secondary outcome measures included the changes in PPD from baseline to 6 months and changes in BOP, REC, IS-AC, IS-BIC, biomarkers and OHIP scores from baseline to 6 and 12 months.

#### Statistics

As this study was an exploratory trial, no power calculation was performed. Statistical analyses were performed using the per protocol population, defined as all randomized patients meeting the inclusion criteria, who completed the trial and who did not have major protocol violations (i.e. no valid primary diagnosis, wash-out period not respected, use of prohibited concomitant medication, less than 6 months of treatment with study medication, randomization code was broken, the patient received wrong study medication, study medication compliance of less than 75%, in- and exclusion criteria not respected, screening failure and medical reason). Results are presented as mean ± standard deviation (SD) in tables and mean with 95% Confidence Interval (CI) in figures. The CBCT parameters, IS-AC and IS-BIC, were analyzed using a linear mixed model with sites nested in implant and patient taken as a random variable. Within group analyses were Bonferroni corrected. All other variables were analyzed by non-parametric tests since the data were not normally distributed. Clinical parameters (PPD and REC), OHIP-scores and biomarker values were analyzed between and within treatments using Mann-Withney U and Friedman with post hoc Bonferroni-corrected Wilcoxon signed-ranks, respectively. BOP, a binary variable, was evaluated using Chi square tests for between-groups analyses whereas differences within treatment groups were analyzed using Cochran’s Q test and post hoc Bonferroni corrected McNemar tests.

A two-tailed p-value below 0.05 was considered statistically significant.

All data were analyzed using SPSS software (version 26.0 for Windows, IBM Corp, Armonk, NY, USA).

## Results

### Patients

**Fig. 2:**
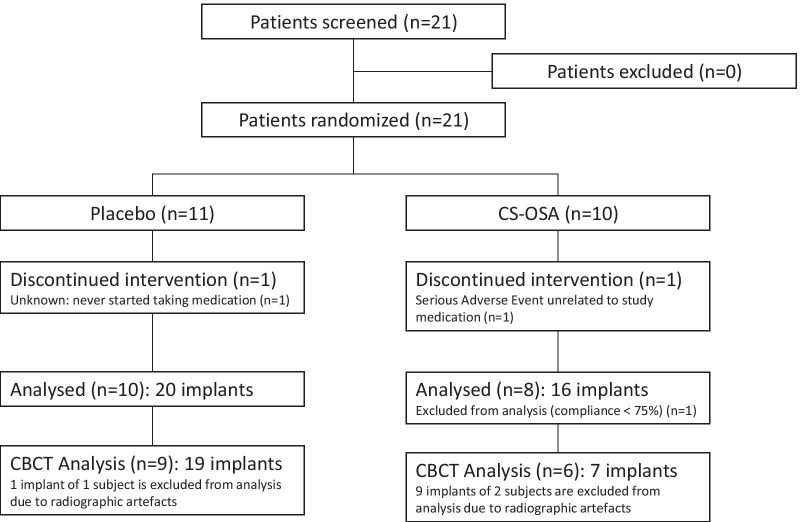
Flow diagram of study enrolment, allocation, follow-up and analysis. CBCT: cone beam computed tomography; CS-OSA: choline-stabilized orthosilicic acid

Between September 2017 and January 2018, a total of 21 eligible subjects were randomized and allocated to receive CS-OSA (n = 10) or a placebo (n = 11). Of these subjects, 5 were successfully treated for chronic periodontitis. No active periodontitis was observed during baseline. There were 4 patients with well-controlled diabetes and 3 of them received CS-OSA. As demonstrated in Fig. 2, data of 18 patients were used in the per protocol analysis. This includes 20 implants in the placebo group and 16 implants in the active treatment group. All implants were tissue level implants. CBCT images of sufficient quality (i.e. without artefacts) to perform analyses were available from 19 implants in the placebo group and 7 in the active treatment group. The study patients’ baseline demographic characteristics are shown in Table 1. No significant differences were found in baseline demographics between the two groups.

**Table 1:** Baseline demographic characteristics and outcome measures

Variable	Treatment group		*p* value
	Placebo	CS-OSA	
Number of patients	10	8	
Number of implants	20	16	
Age	51.50 ± 10.19	52.50 ± 7.29	0.818
Number of males	3	5	0.168
PPD (mm)	5.04 ± 2.37	6.01 ± 2.54	**0.009**
REC (mm)	0.78 ± 1.27	0.70 ± 1.22	0.577
BOP (%)	86.67 ± 34.14	94.79 ± 22.34	**0.045**
IS-AC (mm)#	2.57 ± 2.56	2.4 ± 2.19	0.765
IS-BIC (mm)#	3.83 ± 2.39	4.43 ± 1.98	0.467
OHIP Total	15.10 ± 10.81	14.63 ± 8.35	0.515
hsCRP (mg/L)	2.40 ± 2.40	4.12 ± 2.83	0.055
Osteocalcin (ng/mL)	9.04 ± 3.39	9.88 ± 3.67	0.762
BAP (µg/L)	10.22 ± 4.20	10.13 ± 1.58	0.203
CTX-I (ng/mL)	0.48 ± 0.22	0.52 ± 0.23	0.696
PINP (ng/mL)	43.35 ± 12.61	47.03 ± 8.51	0.573

Data expressed as mean ± SD. Significant difference between groups: *p* < 0.05 = significant (bold) #IS-AC and IS-BIC analysis was performed on cone beam computed tomography (CBCT) scans of sufficient quality (i.e. without artefacts) taken from 9 patients (19 implants) in the placebo group and 6 patients (7 implants) in the CS-OSA group. CS-OSA: choline-stabilized orthosilicic acid, PPD: probing pocket depth, REC: mucosal recession, BOP: bleeding on probing, IS-AC: distance from implant shoulder to alveolar crest, IS-BIC: distance from implant shoulder to first bone-to-implant contact, OHIP: patient’s oral health related quality of life questionnaire, hsCRP: high sensitive C-reactive protein, BAP: bone alkaline phosphatase, CTX-I: C-terminal telopeptide of collagen I, PINP: N-terminal propeptide of type I procollagen

### Compliance and safety

All but one patient reached the minimum compliance of 75%. The mean compliance was 93 ± 8% in the placebo group and 93 ± 6% in the CS-OSA group. One serious adverse event was reported in the active treatment group as the patient passed away due to a heart attack, and was reported by the investigator as unrelated to the study medication. In fact, the analysis of baseline serum samples (i.e. prior of taking study medication) showed that the patient had a high cardiovascular risk as both a high cholesterol level and a high hs-CRP level (> 3.0 mg/L) was found. There were no adverse events reported which were related to the study medication.

### Outcome measures

Clinical parameter outcomes (PPD, REC, BOP) were analyzed at 120 implant sites in the placebo group and 96 sites in the active treatment group. CBCT parameters (IS-AC and IS-BIC) were analyzed at 76 implant sites in the placebo group and 28 in the active treatment group. The baseline outcome measures are summarized in Table 1. Baseline PPD and BOP were significantly lower in the placebo group compared to the active treatment group (PPD: p < 0.01; BOP: *p* < 0.05).

**Fig. 3:**
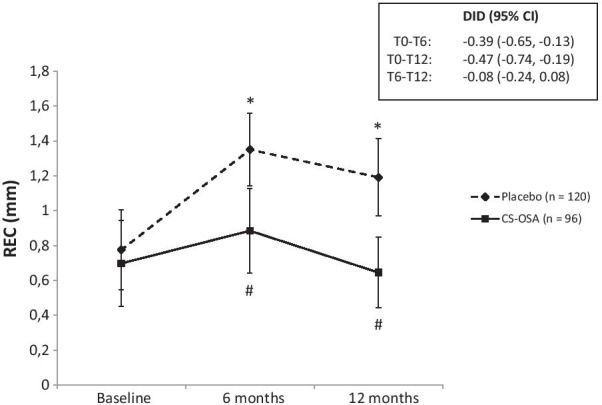
Mucosal recession (REC) at baseline, 6 months and 12 months of treatment with choline-stabilized orthosilicic acid (CS-OSA) compared to placebo. Data is presented as mean with 95% confidence interval (CI). The insert box shows the difference in difference (DID) between baseline and 6 months (T0-T6), baseline and 12 months (T0-T12) and 6 months and 12 months (T6-T12) of treatment with placebo and CS-OSA. *p < 0.05 vs baseline (within placebo); ^#^*p* < 0.05 vs placebo. n = the amount of implant sites

The results of both the primary and secondary outcome measures are summarized in Table 2. PPD and BOP improved significantly (*p* < 0.05) compared to baseline for both groups after 6 and 12 months. However, REC significantly increased in the placebo group (*p* < 0.05) but not in the active treatment group (Fig. 3). The REC value after 6 and 12 months of treatment was significantly higher (p < 0.001) in the placebo group compared to the active treatment group (Fig. 3). Furthermore, the change in REC over 6 and 12 months was significantly different between groups (Table 2). The significant improvement in PPD and BOP after 6 and 12 months was not significantly different between the two treatment groups.

**Table 2:** Primary and secondary outcome measures and the absolute outcome scores

Variable	Treatment group										*p* value	
	Placebo (n = 10)					CS-OSA (n = 8)					*p* value difference in change 6 months	*p* value difference in change 12 months
	Baseline (mean ± SD)	6 months (mean ± SD)	12 months (mean ± SD)	Change 6 months—baseline (mean ± SD)	Change 12 months—baseline (mean ± SD)	Baseline (mean ± SD)	6 months (mean ± SD)	12 months (mean ± SD)	Change 6 months—baseline (mean ± SD)	Change 12 months—baseline (mean ± SD)		
PPD (mm)	5.0 ± 2.4 ^c^	2.8 ± 1.5^a, c^	2.5 ± 1.2 ^a, c^	− 2.2 ± 2.4	− 2.5 ± 2.1	6.0 ± 2.5 ^c^	4.1 ± 2.4 ^a, c^	3.8 ± 2.2 ^a, c^	− 2.0 ± 2.4	− 2.2 ± 2.6	0.320	0.170
BOP (%)	86.7 ± 34.1 ^c^	35.8 ± 48.2^a^	30.8 ± 46.4^a, c^	− 50.8 ± 58.0	− 55.8 ± 54.7	94.8 ± 22.3 ^c^	40.6 ± 49.4^a^	50.0 ± 50.3^a, c^	− 54.2 ± 54.1	− 44.8 ± 54.0	0.691	0.218
REC (mm)	0.8 ± 1.3	1.4 ± 1.2 ^a, c^	1.2 ± 1.2 ^a, c^	0.6 ± 1.0 ^c^	0.4 ± 1.1 ^c^	0.7 ± 1.2	0.9 ± 1.2 ^c^	0.7 ± 1.0 ^c^	0.2 ± 0.9 ^c^	− 0.1 ± 1.0 ^c^	**0.006**	**0.009**
IS-AC (mm)^#^	2.6 ± 2.6	3.0 ± 2.7 ^a^	3.3 ± 2.6 ^a^	0.4 ± 1.0	0.7 ± 1.5	2.4 ± 2.2	2.8 ± 2.2	2.7 ± 2.1	0.4 ± 0.9	0.3 ± 0.9	0.859	0.254
IS-BIC (mm) ^#^	3.8 ± 2.4	4.3 ± 2.3 ^a^	4.6 ± 2.4 ^a^	0.5 ± 1.3	0.8 ± 1.6 ^c^	4.4 ± 2.0	4.7 ± 1.9	4.5 ± 2.1	0.2 ± 0.7	0.1 ± 0.8 ^c^	0.283	**0.023**
OHIP Total	15.1 ± 10.8	14.0 ± 12.6	13.1 ± 7.6	− 1.1 ± 6.8	− 2.0 ± 9.1	14.6 ± 8.4	12.6 ± 8.6	10.1 ± 5.7	− 2.0 ± 10.0	− 4.5 ± 9.8	0.965	0.515
hsCRP (mg/L)	2.4 ± 2.4	2.6 ± 2.6	2.9 ± 1.6	0.2 ± 3.1	0.5 ± 1.6	4.1 ± 2.8	4.0 ± 2.5	3.6 ± 1.8	− 0.1 ± 0.8	− 0.6 ± 3.3	0.829	0.360
Osteocalcin (ng/mL)	9.0 ± 3.4	9.1 ± 2.3	9.4 ± 2.0	0.0 ± 1.9	0.4 ± 3.1	9.9 ± 3.7	9.4 ± 3.1	9.8 ± 4.5	− 0.5 ± 3.4	− 0.1 ± 2.7	0.173	0.696
BAP (µg/L)	10.2 ± 4.2	8.1 ± 3.0 ^a^	10.3 ± 5.9	− 2.2 ± 2.0	0.1 ± 2.2	10.1 ± 1.6	8.2 ± 1.3 ^a^	10.3 ± 2.4	− 1.9 ± 1.2	0.2 ± 2.0	0.829	0.696
CTX-I (ng/mL)	0.5 ± 0.2	0.4 ± 0.2 ^a^	0.5 ± 0.2 ^b^	− 0.1 ± 0.1	0.0 ± 0.1	0.5 ± 0.2	0.4 ± 0.2	0.5 ± 0.2	− 0.1 ± 0.2	− 0.1 ± 0.1	0.515	0.146
PINP (ng/mL)	43.4 ± 12.6	43.2 ± 11.5	43.1 ± 13.2	− 0.1 ± 8.8	− 0.3 ± 6.6	47.0 ± 8.5	48.9 ± 22.8	44.3 ± 18.4	1.9 ± 15.7	− 2.8 ± 12.4	0.897	0.897

Significant difference in changes over 6 and 12 months between groups: *p* < 0.05 = significant (bold). ^a^Significant difference from baseline within the group; ^b^significant difference from 6 months within the group; ^c^significant difference between groups (placebo versus choline-stabilized orthosilicic acid (CS-OSA)). ^#^IS-AC and IS-BIC analysis was performed on cone-beam computed tomography (CBCT) scans of sufficient quality (i.e. without artefacts) taken from 9 patients (19 implants) in the placebo group and 6 patients (7 implants) in the CS-OSA group. CS-OSA: choline-stabilized orthosilicic acid, PPD: probing pocket depth, BOP: bleeding on probing, REC: mucosal recession, IS-AC: distance from implant shoulder to alveolar crest, IS-BIC: distance from implant shoulder to first bone-to-implant contact, OHIP: patient’s oral health related quality of life questionnaire, hsCRP: high sensitive C-reactive protein, BAP: bone alkaline phosphatase, CTX-I: C-terminal telopeptide of collagen I, PINP: N-terminal propeptide of type I procollagen

**Fig. 4:**
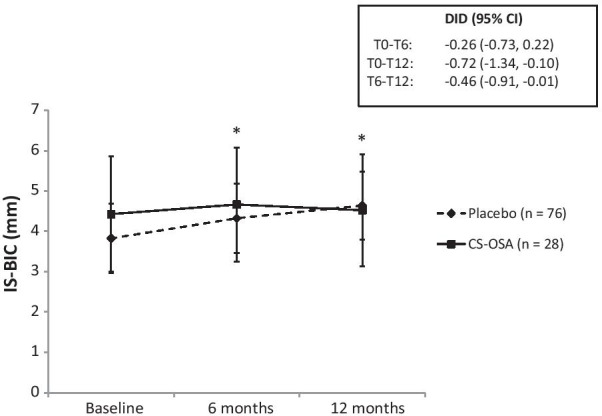
Distance from the implant shoulder to first bone-to-implant contact (IS-BIC) at baseline, 6 months and 12 months of treatment with choline-stabilized orthosilicic acid (CS-OSA) compared to placebo. Data is presented as mean with 95% confidence interval (CI). The insert box shows the difference in difference (DID) between baseline and 6 months (T0–T6), baseline and 12 months (T0-T12) and 6 months and 12 months (T6-T12) of placebo and CS-OSA treatment. *p < 0.05 vs. baseline (within placebo). n = the amount of implant sites

The CBCT parameters IS-AC and IS-BIC significantly increased after 6 and 12 months of treatment in the placebo group but not in the active treatment group. The change in IS-BIC over 12 months was significantly lower in the active treatment group compared to the placebo group (Table 2), resulting in a mean difference (95% CI) between placebo and CS-OSA of − 0.72 mm (−1.34 to −0.10) (Fig. 4). The change in IS-BIC over the last 6 months of treatment (between 6 and 12 months of treatment) was also significantly different between groups with a mean difference (95% CI) of − 0.46 (− 0.91 to − 0.01) (Fig. 4). There were no between group differences observed with respect to IS-AC.

Evaluation of the total OHIP scores indicates no significant improvement in quality of life, neither in the placebo group nor in the active treatment group (Table 2). Biomarker analysis showed no significant differences between the treatment groups (Table 2). With respect to changes in hsCRP levels after 12 months of treatment it is noteworthy to mention that in 70% of the patients in the placebo group the hsCRP levels increased while in the active treatment group increase was only observed in 50% of the patients.

## Discussion

This is the first preliminary study to explore the effect of CS-OSA in patients with peri-implantitis. The main goals of peri-implantitis treatment are resolving inflammation and preventing further bone loss. However, a "gold standard" treatment is still lacking [12]. The clinical parameters PPD and BOP, significantly decreased after 6 and 12 months of CS-OSA and placebo treatment. The observed decreases were not significantly different between groups. We may therefore conclude that there was no significant difference in the primary outcome measure, i.e. the change in PPD at peri-implantitis sites from baseline to 12 months of treatment. The observed decreases in PPD and BOP are in both study groups likely to be the result of the peri-implantitis treatment performed at the baseline visit, i.e. debridement with open flap surgery, followed by 2-monthly repeated oral hygiene instructions. These are common interventions for treating peri-implantitis, which have been previously demonstrated to be effective [12, 26–29]. However, mucosal recession has been reported to be a side-effect of such surgical treatment [30]. Indeed, an increase in REC was observed after 6 and 12 months of treatment in the placebo group, however not in the active treatment group. In fact, the REC value didn’t change during the study period in patients taking CS-OSA suggesting that this complex may have a positive effect on soft tissue healing.

In the present preliminary study, a statistically significant difference between the placebo and the CS-OSA group was observed for bone loss measured by the change in IS-BIC after 12 months of treatment. More specifically, the IS-BIC increased in the placebo group and remained stable in the active treatment group. These findings suggest that CS-OSA may prevent further bone loss at implants with peri-implantitis.

To summarize, a significant increase in both REC and IS-BIC is found after 12 weeks of placebo treatment, while both REC and IS-BIC remained stable in the CS-OSA group. The beneficial effect of CS-OSA on recession might be explained by a direct effect on the gingiva or might be secondary to the beneficial effect of CS-OSA on bone loss, i.e. preventing further bone loss and therefore stabilizing both bone level and the level of the gingival margin.

The effectiveness of CS-OSA on bone and connective tissue health has been previously demonstrated [14, 17, 18, 31]. More specifically, both pre-clinical and clinical studies have shown that CS-OSA has a stimulating effect on the collagen synthesis. Reffitt et al. [14] reported that physiological concentrations of orthosilicic acid stimulate collagen type I synthesis in human osteoblast-like cells and dermal fibroblasts in vitro and promote osteoblastic differentiation. An earlier study in young animals has shown an increase in collagen concentration in the dermis of animals who were fed with CS-OSA in their diet compared to control animals [31]. These reported increases in skin collagen can explain the improved surface and mechanical properties of the skin which were reported after oral intake of CS-OSA in humans [18]. Furthermore, in the study of Spector et al. [17] the use of CS-OSA resulted in an increase of serum PINP and femoral bone density in osteopenic women, indicating improved bone collagen synthesis.

Choline is classified by the Food and Nutrition Board as an essential nutrient and is likely to contribute to the biological activity of CS-OSA [32]. It is a precursor of phospholipids, which are essential components of biological membranes, and is involved in cell signaling and lipid transport/metabolism. One of its metabolites, betaine, participates in the methylation of homocysteine to methionine and therefore reduces the plasma total homocysteine levels [33]. This reduction positively affects collagen cross-linking, since homocysteine has been shown to interfere with post-translational modifications of collagen through direct and indirect inhibition of lysyl oxidase as well as through down regulation of other genes involved in collagen cross-linking [34]. Elevated levels of plasma homocysteine have been detected in patients with chronic periodontitis [35, 36]. These elevated homocysteine levels reduced after periodontal treatment, indicating an important role of homocysteine in periodontal pathologies [36].

The previously described studies ([14, 17, 18, 31, 32, 34–36]) support the hypothesis of a possible effect of CS-OSA on collagen metabolism improving bone and soft tissue healing in patients with peri-implantitis. A positive effect of CS-OSA was indeed confirmed in the present study by the effects observed on IS-BIC and REC, however the biomarker analysis failed to support this hypothesis as no significant differences in serum osteocalcin, PINP, BAP and CTX-I levels were found. These are biomarkers for bone formation and resorption, respectively. This is in contrast with the study of Golub et al. [37] demonstrating reduced serum biomarkers of bone and collagen destruction after periodontal treatment. However, in the latter study, significant effects were observed in the serum of post-menopausal osteopenic women within 5 years of menopause, a time-period associated with high-turnover bone loss, therefore exhibiting not only local but also systemic bone loss. When also taking into account the lack of significant reductions in the inflammatory marker hsCRP in the present study, while the clinical parameter for inflammation, BOP, improved in both treatment groups, it should be encouraged to analyze biomarkers of inflammation, collagen destruction, and bone resorption locally in the peri-implant crevicular fluid. Indeed, an interesting cross-sectional study examining the biomarker profile in peri-implant crevicular fluid from healthy implants and implants with peri-implantitis confirmed that local biomarkers might contribute to distinguish peri-implant health from disease [38]. Also it should be noticed that a large variation was observed in biomarker levels in the present study, which makes it difficult to detect significant differences between groups.

In fact, an important limitation of the present study is the limited number of participants. A limited sample size, without a prior power calculation, was chosen because of the exploratory character of the study. This might explain why the present study failed to show significant effects with respect to the parameters evaluated at patient level, including biomarker and OHIP questionnaire analysis. On the other hand, the significant effects that were found in this study cannot be generalized, but give important insights for future research that should be performed in a larger study population. Another limitation of the study is that not all risk factors for peri-implantitis such as genetic traits, soft tissue quality or quantity, prosthetic design and occlusal overload [7] were standardized between both treatment groups (data unknown). With the linear mixed model, an additional analysis could be performed to investigate the influence of the risk factors “history of periodontitis” and “diabetes” on the outcome results for the bone loss parameters. When history of periodontitis and diabetes were considered as confounders, the significant difference that was found between the placebo and the CS-OSA group for change in IS-BIC after 12 months of treatment remained significant in favor of CS-OSA.

In order to fully understand the mechanisms of action of CS-OSA in preventing further bone loss at the peri-implant site level, as suggested in the present study, it might be useful to investigate biomarkers for bone and collagen metabolism locally in the peri-implant crevicular fluid. As in the present study bone loss was evaluated by linear bone measurements using CBCT, it would be interesting to also look at the effect of CS-OSA on alveolar bone density. Such a study has not yet been performed, since the CBCT technology used in the present study is not the ideal tool for evaluating bone density [39]. The one-year study period in the present trial is a minimum duration to evaluate changes in bone level and/or bone density. In future research, the study period should be prolonged to evaluate the long-term outcomes of the treatment.

## Conclusions

The results of this preliminary study suggest that CS-OSA stabilizes and even prevents further bone loss after surgical peri-implantitis treatment in combination with a beneficial effect on mucosal tissue healing. Future research in a larger, more standardized study population and a longer study period is needed to confirm the results of the present study and to fully understand the exact mechanisms of action.

## Supplementary Information

**Additional file 1.** Dataset Teughels et al. Peri-implantitis study.

## Data Availability

All data generated or analysed during this study are included in this published article and its supplementary information files.

## Abbreviations

2D: Two-dimensional
3D: Three-dimensional
BAP: Bone alkaline phosphatase
BOP: Bleeding on probing
CBCT: Cone beam computed tomography
CS-OSA: Choline-stabilized orthosilicic acid
CTX-I: C-terminal telopeptide of collagen type I
hsCRP: High sensitive C-reactive protein
IR: Intraoral radiography
IS-AC: Distance from implant shoulder to alveolar crest
IS-BIC: Distance from implant shoulder to first bone-to-implant contact
OHIP-14: Patient’s Oral Health related Quality of Life questionnaire
PINP: N-terminal propeptide of type I procollagen
PPD: Probing pocket depth
REC: Mucosal recession
